# Partition Coefficients and Diffusion Lengths of ^222^Rn in Some Polymers at Different Temperatures

**DOI:** 10.3390/ijerph16224523

**Published:** 2019-11-15

**Authors:** Strahil Georgiev, Krasimir Mitev, Chavdar Dutsov, Tatiana Boshkova, Ivelina Dimitrova

**Affiliations:** Faculty of Physics, Sofia University “St Kliment Ohridski”, 5 James Bourchier Blvd, 1164 Sofia, Bulgaria; strahilg@phys.uni-sofia.bg (S.G.); ch.dutsov@phys.uni-sofia.bg (C.D.); boshkova@phys.uni-sofia.bg (T.B.); divelina@phys.uni-sofia.bg (I.D.)

**Keywords:** radon, diffusion, absorption, polymers, polycarbonates

## Abstract

In this work, the partition coefficients *K* and diffusion lengths $L_{D}$ of radon in some polymers are experimentally determined for several temperatures in the range *T* = 5–31 °C. Some of the obtained values are compared to published data available for the given temperatures. It is shown that the temperature dependencies of the partition coefficients K(T), the diffusion lengths $L_{D}\left(T\right)$, and the permeabilities P(T) could be described analytically for the studied temperature range 5–31 °C. This allows estimation of these quantities in the given temperature range and quantitative description of the transport of radon in the studied polymers.

## 1. Introduction

Indoor radon (^222^Rn, half-life 3.8232 d) is recognized as a severe health-risk factor, being the leading cause for lung-cancer after smoking [1]. Due to its short half-life, thoron (^220^Rn, half-life 55.8 s) appears indoors in significant concentrations only under specific circumstances. However, in such cases, thoron could be a health-hazard too [2,3,4]. Therefore, a wide range of methods for radon and thoron measurements are developed dealing with different aspects of the problem—metrological assurance, risk assessment, dose estimation, average activity concentration measurement, mitigation, etc.

Most of the devices used for radon and thoron measurement (either passive or active) are sensitive to both isotopes and some of them are also sensitive to their short-lived progenies (SLPs) [2,3,4,5,6,7,8,9]. The devices able to discriminate between radon, thoron, and SLPs typically use diffusion barrier (incl. polymer membranes) and some are even packed in polymer bags [6,7,8,10,11]. The SLPs are effectively stopped by any diffusion barrier as their atoms are chemically active. On the other hand, radon and thoron are inert gases, and the discrimination between them is based on their different half-lives and the decay during the diffusion through the barrier. The polymer membranes are preferred as they are easy to handle, hydrophobic, flexible, and durable, produced in various thicknesses, and their diffusion properties at room temperature allow good discrimination between radon and thoron. However, the diffusion properties of Rn isotopes in the polymers are temperature dependent. Thus, at a given temperature, the membrane could be almost fully permeable to radon while fully stopping thoron, while, at another temperature, it could be partially permeable to both radon and thoron, which could lead to systematic error in the measurements. The diffusion properties of some polymers are studied in the literature and are quantified by the diffusion coefficient, the permeability, etc. [12,13,14,15]; however, their temperature dependence is rarely mentioned and the obtained values of these parameters vary a lot. A possible reason for these variations could be the different temperatures during the parameters’ estimation. Other reasons that could lead to such variations are related to the production process. It is known that factors such as melting and extrusion temperatures and pressure, cooling speed, presence of some solvents or catalysts have influence on the gas transport properties of the polymers [16]. Additionally, the polymer membranes, depending on their purpose of use, could be compounds of various polymers or polymer layers of different properties and could contain various additives such as fillers, softening agents, UV stabilizers, reinforcements, etc. that could influence their gas transport properties. Therefore, it is important to study the properties of the membranes when the production process (or the producer/supplier) is changed or when they are used under extreme conditions.

Moreover, in the last two decades, it was shown that some polycarbonates such as the Makrofol N and Makrofol DE (Makrofol^®^ family are polycarbonate-based products by Bayer AG, Leverkusen, Germany) have a remarkably high absorption ability to Rn (and other noble gases) [17,18]. Based on that property of the polycarbonates, several methods for radon and other radioactive noble gases (RNGs) measurement were developed. These methods use the polycarbonate as a passive sampler that absorbs and concentrates the RNG from the ambient media. Some of these methods measure the cumulative activity of the absorbed radon relying on the track-etched properties of the Makrofol DE or another (external) track detector [19,20,21], while others register the alpha-, beta- or gamma-particles of the absorbed radon and its SLP (or other RNG) by active detectors—Liquid Scintillation (LS) counters, gross alpha/beta counters, HPGe gamma-spectrometers and others (see [22,23,24,25] and the references there). To apply these methods, the temperature dependence of the diffusion properties of the polycarbonates should be known. This dependence is studied for Makrofol DE [26,27], but, for Makrofol N, the diffusion properties are known only for a single temperature value [23,28].

In [29], it is shown that the transport of RNGs in polymers could be described by two physical parameters: the diffusion length $L_{D}$ of the RNG in the polymer and the partition coefficient *K* of the RNG at the border between the polymer and the ambient media. The purpose of the present work is to estimate experimentally these parameters for radon in some polymers (Makrofol DE, Makrofol N, polypropylene, high-density polyethylene, and low-density polyethylene) at different temperatures and to study their temperature dependence. In the course of the studies, a new approach for precise measurement of the activity of radon in polymers was developed and utilized.

## 2. Materials and Methods

In this work, several polymer foils are studied: Makrofol DE, Makrofol N, polypropylene (PP), high-density polyethylene (HDPE), and two types of low-density polyethylene—plain and anti-slip covered (resp. LDPE and LDPE-A) (The polymer foils PP, HDPE, LDPE, and LDPE-A are supplied from Extrapack OOD, Sofia, Bulgaria.). The choice of the first two is determined by their high absorption ability to RNGs [23,29] and their application for radon measurements. The last four materials are chosen since membranes are made of similar polymers. Such membranes are used for radon/thoron discrimination in some detectors and for radon prevention and mitigation [12].

### 2.1. Transport of RNGs in Polymers

The theoretical model presented and validated in [29] describes the transport of RNG in polymers in two steps/assumptions:

1. The atoms of the RNG are caught in the polymer matrix at the border ambient media/polymer and, in any moment, the ratio of the RNG concentrations at the surface of the polymer $c_{in}$ and, in the ambient media, $c_{out}$ is given by the partition coefficient $K=\frac{c_{in}}{c_{out}}$. It must be noted that the partition coefficient of some polymers could be greater than one (For example, K≈100 for ^222^Rn at the border Makrofol N/air at room temperature, which makes it very appropriate for a radon sampler). One possible explanation of this phenomenon could be the presence of free-volume traps in the polymer matrix (see [28] and the references there). In the free-volume trap models, it is considered that there are small voids in the polymer matrix with sizes close to the dimensions of the RNG atoms. The RNG atoms are trapped in these voids, and the concentration of the RNG in the polymer appears to be higher than in the ambient media;
2. Once the RNG atoms are caught in the polymer matrix, their transport in the polymer is described by the diffusion equation (Fick’s second law) with an additional term that accounts for the radioactive decay:
  (1) $$\frac{\partial c}{\partial t}=D\left(\frac{\partial^{2}c}{\partial x^{2}}+\frac{\partial^{2}c}{\partial y^{2}}+\frac{\partial^{2}c}{\partial z^{2}}\right)-\lambda c,$$
  where c(x,y,z,t) [m^−3^] is the RNG concentration in the polymer sample as a function of the space x,y,z [m] and time *t* [s] coordinates (Hereafter, the units of the quantities according to the Intentional System of Units (SI) are given in square brackets “[ ]”, when the quantity is introduced for the first time in the text), *D* [m^2^/s] is the diffusion coefficient of the atoms of the noble gas in the polymer, and λ [s^−1^] is the decay constant of the RNG. In [29], Equation (1) is solved for some given shapes of the polymer samples, immersed in RNG-containing media. Once the polymer sample is exposed, it absorbs the RNG, and the dynamics of the absorption depends on the exposure conditions, polymer geometry, and on the parameters *K* [dimensionless] and *D*. In the present work, plate-shaped specimens are considered exposed to radon in air for time $t_{s}$ [s] and left to desorb in infinite radon-free media for time $t_{d}$ [s]. In the considered exposure, radon is promptly introduced in the exposure volume and then the activity concentration of radon decreases exponentially (due to radioactive decay) with the decay constant of radon. For plate-shape specimens (specimens for which one of the dimensions is orders of magnitude smaller than the others), the process is considered one-dimensional, and the solution for the RNG activity $A(t_{s},t_{d})$ [Bq] absorbed in the specimen is [29]:
  (2) $$A\left(t_{s},t_{d}\right)=\frac{8\lambda L_{D}^{2}VKC_{A}}{L^{2}}\sum_{k=0}^{\infty }\frac{e^{-\lambda t_{s}}-e^{-\lambda_{k}t_{s}}}{\lambda_{k}-\lambda }e^{-\lambda_{k}t_{d}},$$
  with
  (3) $$\lambda_{k}=\lambda \left(1+{\left(\frac{\left(2k+1\right)\pi L_{D}}{L}\right)}^{2}\right),$$
  where *L* [m] and *V* [m^3^] are the thickness and the volume of the specimen, $C_{A}$ [Bq/m^3^] is the initial activity concentration of the RNG in the media, and $L_{D}$ [m] is the diffusion length of the RNG in the polymer. In this model, the only two parameters are the partition coefficient *K* and the diffusion length $L_{D}$. The latter is by definition related to the diffusion coefficient *D*: $L_{D}=\sqrt{D/\lambda }$. Thus, if the two parameters *K* and $L_{D}$ (or *D*) are known, the transport of the RNG in/through a polymer membrane could be quantitatively described. It must be noted that Equation (2) is derived for the more general case of transient radon distribution in the sample and is valid for arbitrary sorption and desorption times. The only restrictions to Equation (2) are the plate shape of the specimens and the exponentially decreasing ambient activity concentration (In [29], Equation (1) is also solved for constant ambient activity concentration and for cylindrical specimens).

### 2.2. Method for Estimation of *K* and $L_{D}$

Based on the above-described model, a method for estimation of *K* and $L_{D}$ is developed [30] and later modified [23]. In the modified method, several identical plate-shaped polymer specimens are exposed in RNG-containing media under controlled conditions. The specimens are then left to desorb in RNG-free media, and each one is submerged in an LS cocktail and measured by LS counting at a different moment after the exposure, in order to study the decrease of the absorbed activity due to decay and desorption. The obtained time-dependence $A(t_{d})$ is fitted with the theoretical function given by Equation (2). For that purpose, it is more convenient to combine Equations (2) and (3) in the following way:

(4) $$A\left(t_{d};K,L_{D}\right)=8VC_{A}K\sum_{n=1}^{\infty }\frac{e^{-\lambda t_{s}}-e^{-\lambda \left(1+{\left(n\pi \right)}^{2}{\left(\frac{L_{D}}{L}\right)}^{2}\right)t_{s}}}{{\left(n\pi \right)}^{2}}e^{-\lambda \left(1+{\left(n\pi \right)}^{2}{\left(\frac{L_{D}}{L}\right)}^{2}\right)t_{d}},$$

where n=2k+1 is an odd number. Since the exposure conditions and the specimen dimensions are known, the only unknown (free) parameters in Equation (4) are *K* and $L_{D}$. The infinite sum in Equation (4) is convergent and it converges faster with the increase of $t_{d}$ and the ratio $L_{D}/L$. Thus, after a certain time of desorption $t_{d}$ (depending on the ratio $L_{D}/L$), the sum could be restricted to a reasonable number of terms *n* and *K* and $L_{D}$ could be estimated by fitting the experimental data for $A_{i}\left(t_{d,i}\right)$ with the model curve $A(t_{d};K,L_{D})$ from Equation (4). An important advantage of the method is that it is applicable in transient (non steady-state) conditions. The only restrictions to it are: (1) The specimens have to be plate-shaped and the ambient activity has to decrease exponentially (see Section 2.1) and (2) $L_{D}/L$>0.2, preferably $L_{D}/L$>1, so that the convergence of the series in Equation (4) is faster. In all experiments presented in this manuscript, these restrictions are obeyed.

### 2.3. Measurement of the Absorbed Activity

For the activity follow up, two approaches are considered. The first is direct LS measurement of the absorbed activity. In [23], it is shown that Makrofol N is soluble in a toluene-based LS cocktail. In the present work, the same toluene-LS cocktail is used for the Makrofol N measurements: high performance glass vials with a foil-line cap by PerkinElmer (Waltham, MA, USA) are fully filled with the toluene-LS cocktail and the Makrofol N foils are periodically immersed in it. Once the foils are closed in the vials, they are dissolved in the LS cocktail—the activity is fully transferred in the cocktail and, when equilibrium is reached between radon and its SLP (after 4h), the activity in the vials decreases with the half-life of radon. Makrofol N fully dissolves in a toluene-based cocktail, which allows the absorbed activity to be measured with a common LS counter or via absolute measurement with a TDCR-counter [23] (TDCR—Triple to Double Coincidence Ratio).

On the other hand, the Makrofol DE foil is only partially soluble in toluene-based cocktail (some fine particles remain) while the other four polymers are insoluble in toluene. This could lead to variations in the measurement efficiency, since the RNG partially desorbs from the specimen to the LS cocktail during the measurement. The change in efficiency due to the different distribution of activity in the specimen and the cocktail could be significant especially for alpha-particles: an alpha-particle emitted in the scintillator is detected with 100% detection efficiency [31] while one emitted in the volume of the polymer will be detected only if it reaches the scintillator. Some other organic solvents—Gasoline, Bensol (Benzene), 1,2Dichloroethane (ethylene dichloride) were also tested and they did not dissolve PP, HDPE, and LDPE—despite the fact that the storage of some of these solvents in PE bottles is not allowed, suggesting they should react with PE. Further discussions with chemists confirmed that PE is somewhat resistive to lots of chemical solvents. Therefore, the second approach chosen in the current work is Cherenkov-counting of the polymer by LS-counter. A similar approach for direct Cherenkov-counting of RNGs absorbed in Makrofol DE grains is presented in [32]. The basic idea is to place the Makrofol DE grains in LS-vial and to register the Cherenkov light (e.g., with a common LS-counter) emitted by the beta-particles (of the SLP of ^222^Rn) passing through the Makrofol DE. However, the polymer foils used in the present work are thin, which leads to very fast desorption of radon from the specimen to the air in the empty LS-vial. This could lead to a change in the counting efficiency and a loss of radon from the vial that could not be followed and corrected for. This is why the approach was modified: the LS-vials are fully filled with distilled water, and the polymer specimen is immersed in it. When the polymer foil is immersed in the water, some of the radon absorbed in the foil is released in the water until equilibrium between the radon concentration in the two media is reached. The equilibrium is determined by the partition coefficient at the border water–polymer. During the process of redistribution of the activity, the Cherenkov counting efficiency changes as the Cherenkov effect depends on the refraction index of the media. Once equilibrium is reached, the efficiency is constant (as it is shown further in this work), and it could be used to determine the activity in the sample.

The two approaches (LS and Cherenkov counting) were chosen due to several advantages they offer, compared to gamma-spectrometry or external gross beta-counting:

- These approaches allow precise timing—when the foil is closed in the vial, the activity is “trapped” in the vial, thus it could be attributed to the exact moment of desorption within 1–2 s.
- There is a small (for the Cherenkov) or even no (for the LS) activity leakage from the vials (see further in Section 3.1). Thus, if the samples have to be measured later or for a longer time, the activity will be sufficient for a longer time and precise long measurements can be performed.
- As the activity is “trapped” in the vial, there is no need for temperature control during the measurement. In the case of gamma-spectrometry and external gross counting, the samples have to be kept at the studied temperature; otherwise, the desorption will be compromised. This is inconvenient or even unachievable in the case of a temperature that differs with more than 5–10 °C from the normal room temperature.

## 3. Experiments

Two series of experiments were carried out. In all experiments high-performance glass vials with foil-lined caps (Perkin Elmer, Waltham, MA, USA) were used. All polymer foils were cut in the same rectangular shape 1.60(5) cm × 5.70(5) cm (The uncertainties of the quantities reported hereafter are given in parenthesis according to [33]) in order to fit reproducibly in the glass vials. The thicknesses of the foils were measured with micrometer with 1 µm instrumental uncertainty. For the measurements of the activity in the foils, three detectors were used: a TDCR-counter [34], a HPGe detector with relative efficiency 24.9 %, and resolution 1.9 keV for the gamma-line 1332 keV of ^60^Co (ORTEC, Oak Ridge, TN, USA) and an LS-analyzer RackBeta 1219 (Wallac, Turku, Finland). For the measurements of the activity concentration of radon in air during the exposure a reference monitor AlphaGUARD RnTn Pro (Saphymo, Frankfurt, Germany) was used.

### 3.1. Estimation of the Counting Efficiencies

The first series of experiments was dedicated to the estimation of the counting efficiency $\epsilon_{c}$ of the RackBeta 1219 LS-counter for LS-counting of Makrofol N in the toluene LS-cocktail and for Cherenkov counting of polymer foils in water. In the case of Makrofol N in LS-cocktail, a foil was exposed to radon; then, it was dissolved in the toluene cocktail and measured on the LS-counter and on the TDCR-detector. The TDCR allows absolute measurement of the activity in the vial [23], and the efficiency was estimated as the ratio of the counting rate of the LS-counter and the activity in the vial. The obtained value was $\epsilon_{c}=$ 4.946(29). Note that this is the efficiency for radon in equilibrium with its SLP, i.e., 5 particles (the 3 alphas of ^222^Rn, ^218^Po, ^214^Po and the 2 betas of ^214^Pb, ^214^Bi) are emitted per one decay of radon.

**Figure 1:**
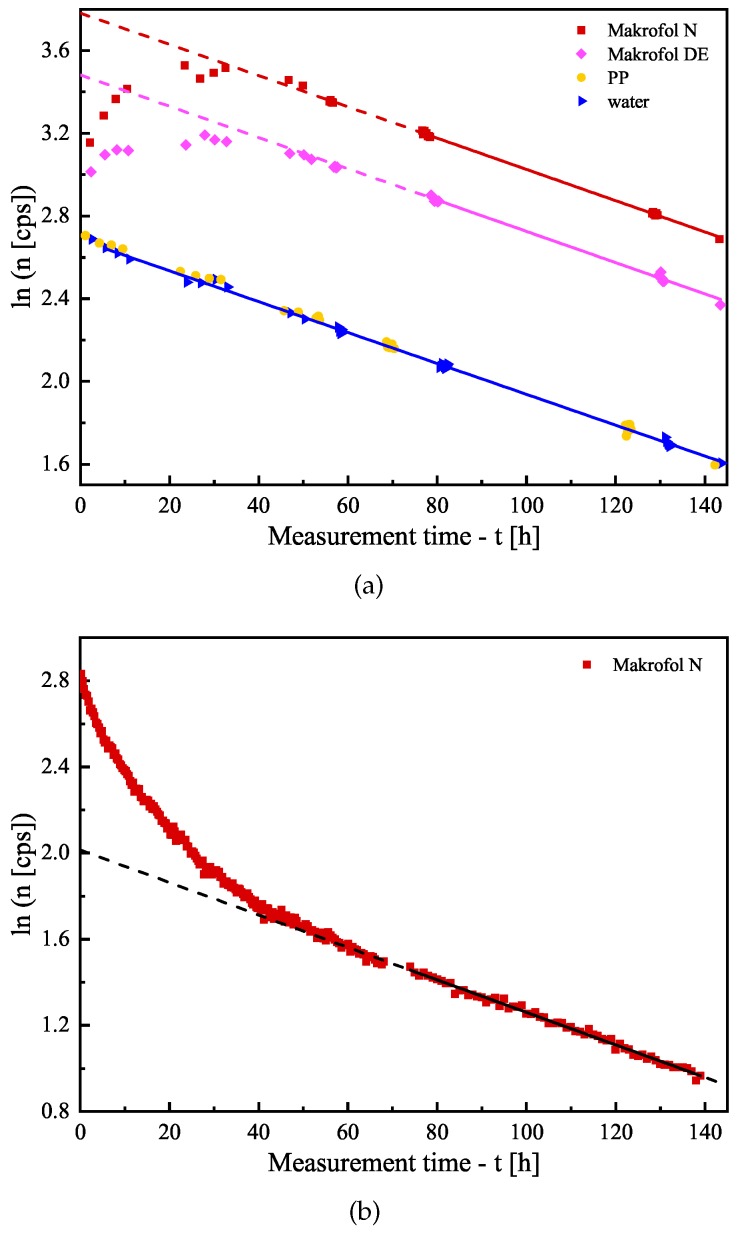
Signal follow-up (in semi-logarithmic scale) of several samples measured at the liquid scintillation counter in Cherenkov-counting mode: (**a**) unexposed polymer foils immersed in water with radon activity and (**b**) a Makrofol N foil exposed to radon immersed in distilled water. The points are the experimental data (the uncertainties—not shown, are within the size of the symbols), the solid line is a linear fit of the data, and the dashed line is extrapolation of the fit for better visualization. The signals decrease linearly in semi-log. scale (i.e., exponentially) and the slopes are very close.

For the estimation of the counting efficiency of the polymer foils in water, spring water from the town of Momin Prohod, Bulgaria with high radon concentration (about 2 MBq/m^3^) was used. Glass vials were fully filled with this water, unexposed foils were placed in the vials, and the vials were closed tightly. Two vials with water without foils were also prepared for comparison. Then, all the vials were periodically measured on the LS counter in order to follow the signal change in time. The duration of a single measurement was 10 minutes, and the whole follow-up experiment continued for about one week. The vials were also measured at the HPGe detector (2–3 measurements of each vial with a few hours duration) in order to estimate the activity in the vials, thus to estimate the counting efficiency of the LS counter. For the gamma–spectrometry analysis, the 295 keV and 352 keV gamma-lines of ^214^Pb were used. The experiment was carried-out contrariwise—unexposed foil in water with activity, instead of exposed foil in distilled water, in order to ensure better counting statistics, thus, to be more sensitive to slight changes in the signal due to the redistribution of radon between the water and the foils. The follow-up measurements at the LS-counter show that the signal of all samples, except those with Makrofol foils, decreases purely exponentially with the same (statistically) effective half-life as the signal of the distilled water samples (see, for example, Figure 1a). The average value of the effective half-life is 3.728(36) d, which is slightly lower than the radon half-life of 3.8232(8) d [35]. It was also observed that, in the first 60-70 h of the follow-up, the signal from the samples with the two types of Makrofol foils increases, reaches a maximum and then starts to decrease and, after 60–70 h, the decrease becomes exponential with the same above-mentioned effective half-life (see Figure 1a). The initial increase of the signal could be explained by the absorption of radon in the Makrofol foils—these foils absorb a significant part of the radon from the water. Due to their higher refraction index ∼1.6 [36] (compared to that of the water 1.33), they have higher efficiency for Cherenkov light emission (the higher the refraction index is, the lower is the threshold energy for the beta-particles to produce Cherenkov effect). Additionally, the Makrofol material possesses some (poor) scintillation properties [36], which also might lead to increasing the counting efficiency.

The activity measurements with the HPGe show that the signals of all samples decrease with the same (statistically) effective half-lives that coincide with the average half-life obtained for the LS-counting. This leads to a few conclusions: the samples are almost hermetic to radon with a small leakage of radon that could be accounted for; the effect of the redistribution of radon between the water and the polymer is significant only for the two types of Makrofol; for all samples, the counting efficiency becomes constant, after a certain period of time—for PP, LDPE, LDPE-A, and HDPE, this period is 3–5 h (the time needed for reaching secular equilibrium between radon and its SLP) and, for Makrofol N and Makrofol DE, this period is about 60–70 h (the time needed for radon redistribution and reaching equilibrium in the two phases—polymer–water).

In this series, one more experiment was carried out: a Makrofol N foil was exposed to radon and then immersed in an LS glass vial full with distilled water—in the same way, the further experiments on *K* and $L_{D}$ estimation are made. The purpose was to check if it matters for the counting efficiency in which direction the activity redistribution between water–polymer goes. In this experiment, only Makrofol N foil was used for two reasons: first, Makrofol N has the highest absorption ability, so it is the best for the counting statistics and, second, the change of the signal due to the redistribution is most pronounced for Makrofol N. Again, the signal from the foil was followed by the LS-counter (see Figure 1b) and measured several times at the HPGe detector. In this case, the LS-counting shows a faster decrease of the Cherenkov signal (due to desorption of the activity from the Makrofol N foil in the water, thus the Cherenkov efficiency decreases) in the first 60–70 h and, then, after equilibrium is reached between the radon in the two phases polymer–water, the signal decrease becomes exponential as in the previous experiment. Again, the gamma–spectrometry shows a single exponential decrease for the entire time of the follow-up. Additionally, the Cherenkov counting efficiencies as a function of time $\epsilon_{c}\left(t\right)$ [dimensionless] were estimated for all foils in the two experiments using the net LS-counting rate $n_{0}\left(t\right)$ [s^−1^] and the gamma-spectroscopically measured activity in the sample A(t):

(5) $$\epsilon_{c}\left(t\right)=\frac{n_{0}\left(t\right)}{A(t)}.$$

**Figure 2:**
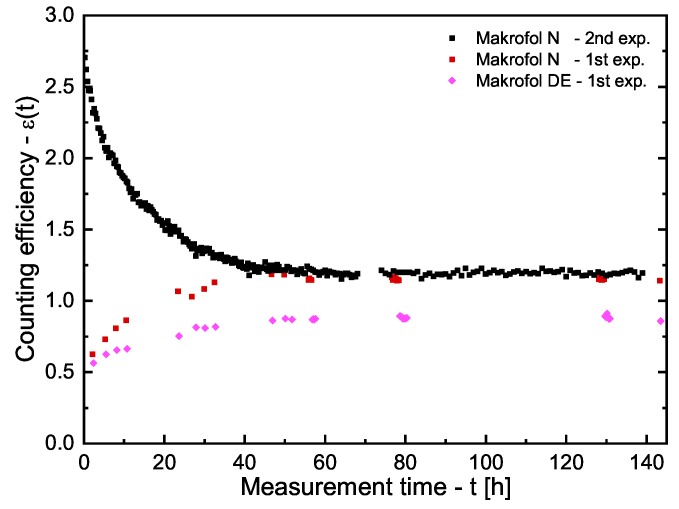
Cherenkov-counting efficiencies as a function of time for the two types Makrofol foils immersed in water. The uncertainties (not shown) at the level of 1σ are about 5% for the points from the first experiment and about 3% for the points from the second experiment.

For the PP, LDPE, LDPE-A, and HDPE, no time-dependence of $\epsilon_{c}\left(t\right)$ was observed. The obtained dependence of $\epsilon_{c}\left(t\right)$ for the Makrofol DE and Makrofol N (both experiments) is shown in Figure 2. It is seen that, after 60–70 h, the counting efficiencies become constant and, for the Makrofol N foils from the two “Cherenkov-counting” experiments, the counting efficiencies are in excellent agreement. These observations lead to the conclusion that the Cherenkov-counting efficiency does not depend on the initial distribution of radon in the two phases. The counting efficiencies obtained in these series of experiments are given in Table 1. It should be noted that the Cherenkov-counting efficiencies of PP, LDPE, LDPE-A, and HDPE are very close to each other and to that of the pure water, while those of the Makrofol foils are significantly higher. This could be due to the much lower partition coefficients of the first four polymers compared to the partition coefficients of the Makrofol foils (see below) or due to the weak scintillation properties of the polycarbonate. Rough estimates show that, when equilibrium of radon between the two phases is reached, the activity of radon in the first four polymer foils used in this work is less than 1% of the total activity in the vial, while, in the Makrofol DE, it is about 15% and in Makrofol N—about 30%.

### 3.2. Estimation of *K* and $L_{D}$

**Figure 3:**
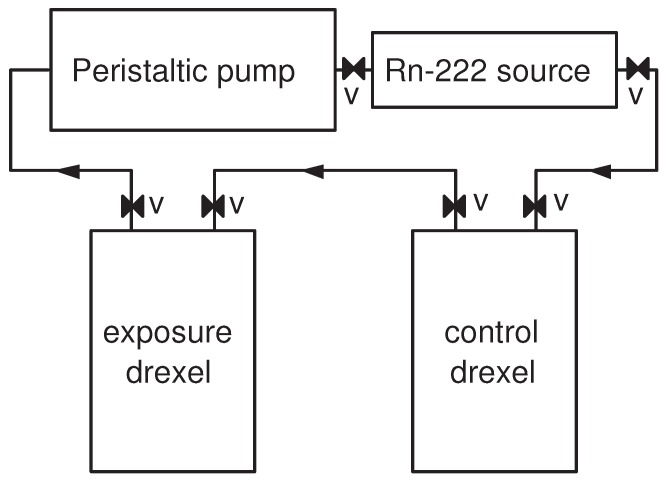
A scheme of the exposure system. In the beginning of the exposure, the activity of the radon source was promptly introduced in the system by the pump. Then, the valves “V” were closed, and the system was disconnected.

The second series of experiments was dedicated to estimation of the partition coefficient and the diffusion length of radon in the studied polymers at different temperatures. Four experiments were carried out at four different temperatures. In these experiments, the foils were exposed to known radon concentration in air. In the first three experiments, six rectangular pieces of each type of foil (36 pieces in total) with dimensions 1.6 cm × 5.6 cm × *L* (the thickness of the foils *L* is measured by a digital micrometer with 1 µm resolution) were stacked in a holder and placed in a hermetic “exposure” drexel. The “exposure” drexel (700 mL) was connected in a closed loop with ^222^Rn source (≈100 kBq, ≈200 mL), a peristaltic pump, and another “control” drexel (700 mL) (see Figure 3). The radon activity was promptly introduced in the system by opening all valves and turning on the pump at 2 L/min flow-rate for 5 min. After that, all valves were closed and the foils were exposed for 2–3 days. Thus, the exposure activity concentration in these three experiments was of the order of tens of MBq/m^3^. Such high activity concentration was needed to ensure good counting statistics for the follow-up of the foils. During the exposure, each drexel was placed in a bigger hermetic vessel, and the radon concentration in the bigger vessels was measured by the AlphaGUARD. This was done in order to check for radon leakage from the drexels. In all experiments, the leakage from the drexels was found to be less than 1% of the radon activity in the drexel. During the exposure, the bigger vessel (with the “exposure” drexel inside) was placed in a thermostat [37] and the exposure temperature was kept stable within 1 °C. The “control” drexel was used for estimation of the activity concentration during the exposure: Because the exposure activity concentration was above the measurement range of the AlphaGUARD, the activity from the “control” drexel was diluted in a larger vessel with a well-known volume. Thus, the activity concentration in the larger vessel was lowered to the measurement range of the AlphaGUARD—it was measured, and the initial (exposure) activity concentration was calculated based on this measurement and the volume ratio of the drexel and the larger vessel. The exposure data are summarized in Table 2.

The fourth experiment was carried out in a 50 L calibration container, which is a part of the AlphaGUARD set. In this experiment, the activity concentration was lower (not enough to obtain measurable signal from all polymers) and therefore only Makrofol foils were exposed. In this experiment, seven pieces of Makrofol DE and twelve pieces of Makrofol N with the same dimensions as in the previous experiments were used. The activity concentrations during the exposure were measured by the AlphaGUARD monitor. The exposure vessel was placed in the hermostat [37], and the exposure temperature was kept stable within 1 °C. The exposure data are shown in Table 2.

After the exposure, the desorption of the radon absorbed in the foils was followed according to the procedures described in Section 2.3. Then, the method described in Section 2.2 was applied on the obtained time dependences in order to estimate *K* and $L_{D}$.

## 4. Results

At the end of the exposure, for all experiments in the second series (*K* and $L_{D}$ estimation series), the foils were removed from the exposure vessel and left to desorb in radon-free air. The temperature of the air was kept the same as the one during the corresponding exposure. Periodically, a foil of each type was immersed in an LS vial filled with distilled water or toluene LS cocktail (in the case of Makrofol N). The time intervals between the immersion of the foils of each type were optimized according to the desorption speed and varied from one minute to 10–15 hours. This optimization aimed to balance between the following two factors:

1. The uncertainties of the individual points of the desorption follow-up. We aim to achieve relative uncertainty of the net counting rate comparable to or better than that of the counting efficiency (see Table 1), i.e., a few percent;
2. The change (decrease) of the absorbed activity due to the desorption. The model curve (see Equation (4)) is a sum of several exponents in which the quantities *K* and $L_{D}$ are parameters. In order to achieve a better estimate of the parameters, it is important to observe greater differences in the activity in the sample, i.e., to follow the desorption for a longer time. However, this leads to a decrease in the counting rate and an increase in its statistical uncertainty.

**Figure 4:**
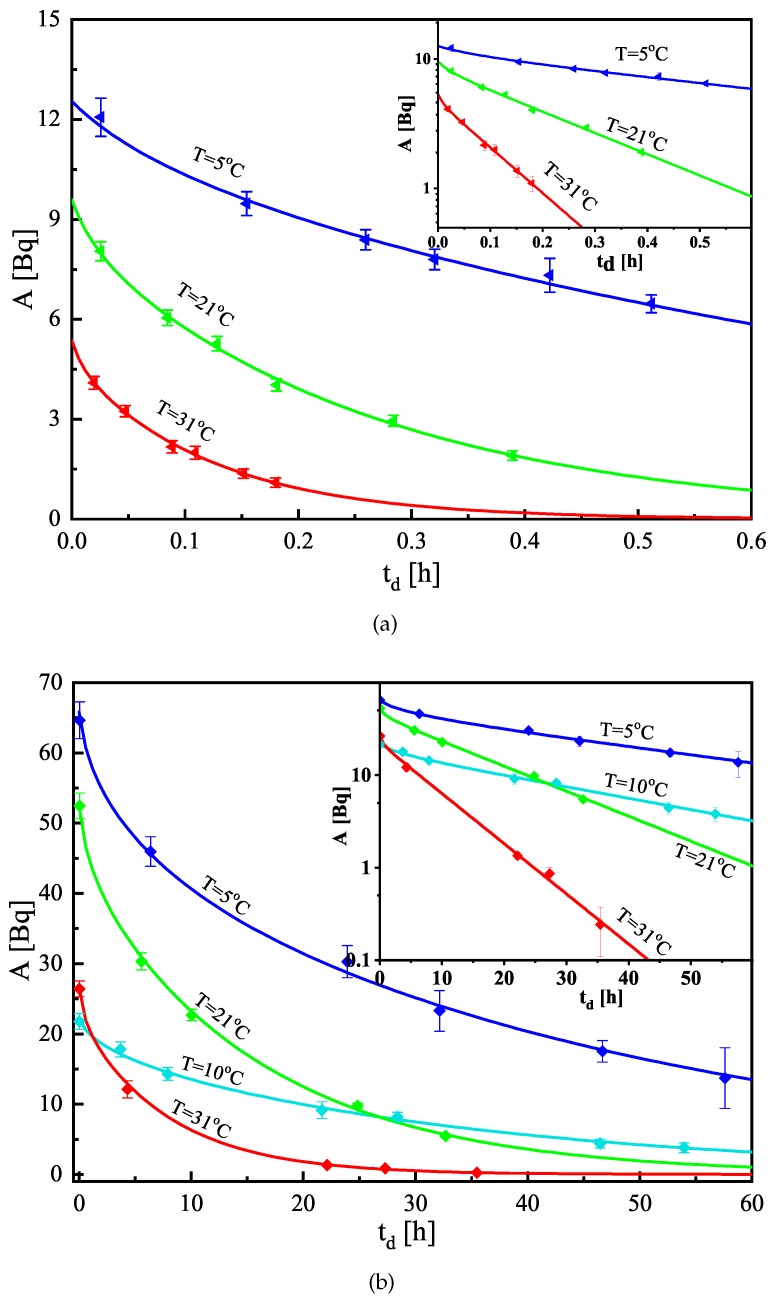
Experimental data (points) and theoretical curve fits (solid lines) of the desorption follow-up of radon from (**a**) High-density polyethylene and (**b**) Makrofol DE foils for the estimation of the partition coefficient and diffusion length at different temperatures. To fit the same scale, the activity data of Makrofol DE at 10 °C are multiplied by 10, as the radon activity concentration in this experiment was one order of magnitude lower than in the other three. The uncertainties are at the level of 1σ. The embedded smaller graph presents the same data in semi-log scale—it is seen that, in the early desorption the dependences are nonlinear in semi-log scale, i.e., they are sums of several exponents rather than single exponents.

When the foils were placed in the LS vials, they were measured consecutively on the LS-counter. After the time needed to reach equilibrium (see Section 3.1), the obtained LS-signal was plotted in a semi-logarithmic scale (similarly to the data shown in Figure 1), and the data after the equilibrium were used to estimate the net counting rate at the moment the foil was placed in the vial. The obtained net counting rate and the counting efficiencies were used to estimate the activity in the foil at the moment of its placement in the LS-vial. In this way, the combination of the individual activities of the foils of given material constructs a very precise follow-up curve of the desorption of radon from the different materials. The obtained desorption data were fitted with the model curve (For the nonlinear curve fitting, the Levenberg–Marquardt optimization algorithm [38] is used. The uncertainties of the experimental points were used as weights for the fitting.) following Equation (4) and the partition coefficient and the diffusion length were estimated from the fit (see Table 3). An example of the fitting is shown in Figure 4.

Additionally, in the fourth experiment in which twelve Makrofol N were exposed, six of them were measured in water and six—in toluene LS cocktail. This was done in order to compare the two measurement approaches. The results obtained for both *K* and $L_{D}$ are in very good agreement within the uncertainties.

For comparison, the values of *K* and $L_{D}$ for some of the materials obtained in previous studies are also shown in Table 3. It is seen that all of them except Makrofol N show significant differences. The differences are even more pronounced when comparing *K* and $L_{D}$ for Makrofol DE at different temperatures. It should be noted that only Makrofol N is physically the same foil—all Makrofol N foils used in the current and the previous study are cut from a single (larger) sheet. All other foils are different (including from different producers), even though the materials (chemical compound) are the same. A very probable reason for that could be the differences in the process of production of the foils e.g., melting and extrusion temperatures and pressure, the presence of some solvents or catalysts, cooling speed, etc. This implies that the production process has an effect on the diffusion properties of the polymers [16].

**Figure 5:**
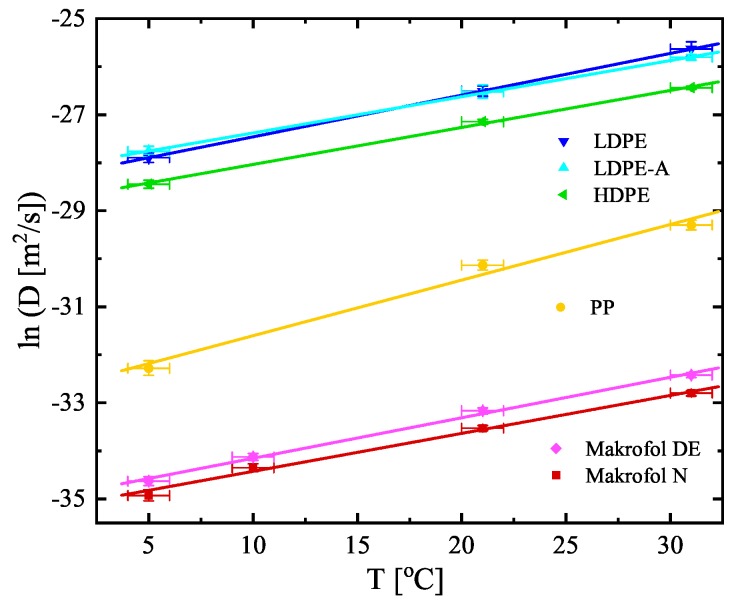
Temperature dependence of the diffusion coefficients of the studied materials. Note that the dependence is ln(*D*) vs. *T*. The points are the experimental data and the solid lines are linear fits of the data. The uncertainties are at the level of 1σ.

**Figure 6:**
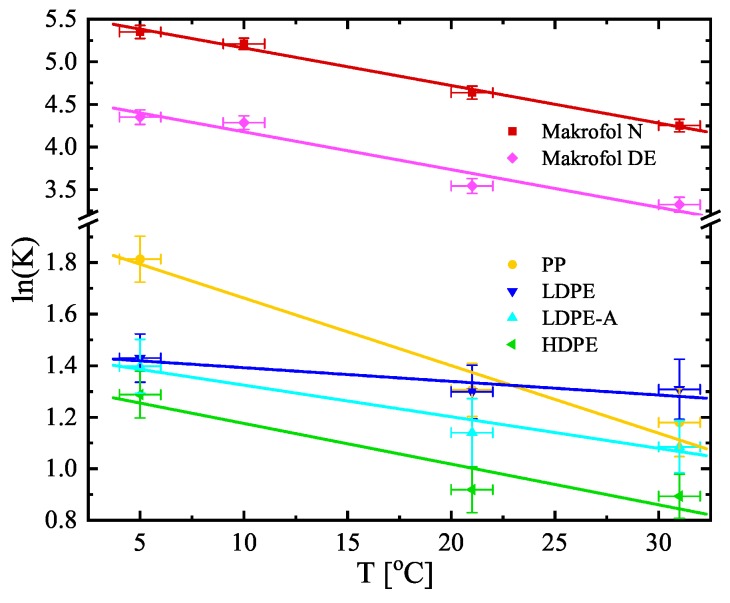
Temperature dependence of the partition coefficients of the studied materials. Note that the dependence is ln(*K*) vs. *T*. The points are the experimental data and the solid lines are linear fits of the data. The uncertainties are at the level of 1σ.

**Figure 7:**
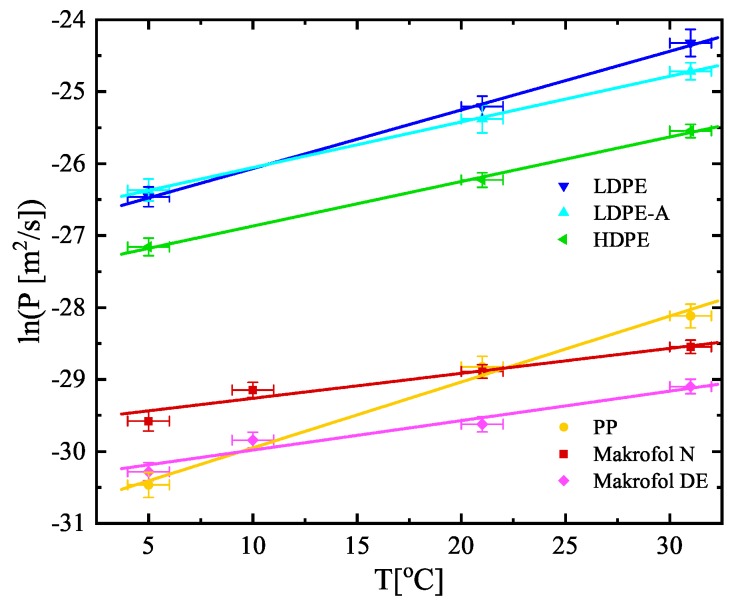
Temperature dependence of the permeabilities of the studied materials. Note that the dependence is ln(*P*) vs. *T*. The points are the experimental data and the solid lines are linear fits of the data. The uncertainties are at the level of 1σ.

The estimated values of *K* and $L_{D}$ (shown in Table 3) are used to calculate the other two quantities, often used to describe the transport of radon through polymer membranes—the diffusion coefficient $D=\lambda L_{D}^{2}$ and the permeability P=KD [m^2^/s] (also shown in Table 3). The diffusion coefficients, the partition coefficients, and the permeabilities versus temperature are also shown in Figure 5, Figure 6 and Figure 7 (the diffusion lengths are not shown as $L_{D}=\sqrt{D/\lambda }$). It is seen that their temperature dependences could be described analytically for the studied temperature interval (5–31 °C). The parameters of the linear fits shown in Figure 5, Figure 6 and Figure 7 are summarized in Table 4. That allows for estimating the values of the quantities for a given temperature in that interval and thus to model the absorption and transport of radon in the polymers.

## 5. Conclusions

In the present work, the temperature dependence of the physical parameters (diffusion length, partition coefficient, diffusion coefficient, and permeability) that describe the transport of radon through some polymers are studied. The values of these parameters are determined at several temperatures in the interval 5–31 °C and their temperature dependences are modeled analytically. Significant temperature dependence of the parameters for all polymers is observed. The knowledge of the temperature dependences of the parameters and the possibility to model those dependencies analytically allow for predicting the behavior of the polymers at different temperatures, which would facilitate their various applications (e.g., radon/thoron discrimination, radon mitigation, radon sampling, etc.). The results reported in this work allow for modeling radon transport in polypropylene, low- and high-density polyethylene, Makrofol N and DE polycarbonates in the temperature range 5–31 °C.

The estimated values of the diffusion lengths and the partition coefficients are compared with such from previous studies of materials declared as chemically the same. Significant discrepancies are observed for all of the compared materials except for Makrofol N. Discrepancies are observed even for the two LDPE materials (LDPE and LDPE-A) from the current study (the LDPE-A material is LDPE from the same producer with anti-slip coating). These discrepancies are attributed to differences in the production process of the polymers. Therefore, it is recommended to test the properties of the specific material from the selected producer including at the extreme exploitation temperatures.

In addition, a new method for radon-in-polymer measurement is proposed. In this method, a thin polymer foil that already is exposed to radon is immersed in an LS-vial fully filled with distilled water. The vial is closed and measured by a standard LS-analyser. The beta-particles of the short-lived progeny of radon emit Cherenkov light in the water, which is detected by the LS-analyser. The method is very appropriate for studies of the RNG-transport properties of polymers, especially when the transport process is fast, as it allows precise timing, long duration of the measurements (with decay correction), and it does not require temperature control during the measurement.

## Figures and Tables

**Table 1:** Counting efficiencies for: polymer foils in distilled water counted in Cherenkov mode (1–6), distilled water counted in Cherenkov mode (7) and Makrofol N foil dissolved in toluene based liquid scintillation cocktail (LSC) (8). The counting efficiencies are given after reaching equilibrium distribution between radon concentration in the two phases (polymer–water) and/or equilibrium of radon and its short-lived progeny, i.e., these are steady-state counting efficiencies. For (1–4, 7,8), equilibrium is reached after 3–5 h and for (5,6) equilibrium is reached after 60–70 h.

No	Sample	Counting Efficiency
1	PP in water	0.380(12)
2	LDPE in water	0.371(12)
3	LDPE-A in water	0.400(14)
4	HDPE in water	0.407(13)
5	Makrofol N in water	1.168(36)
6	Makrofol DE in water	0.883(29)
7	distilled water	0.376(12)
8	Makrofol N in LSC	4.946(29)

PP – Polypropylene, LDPE – Low-Density Polyethylene, LDPE-A – Low-Density Polyethylene with Anti-slip coating, HDPE – High-Density Polyethylene.

**Table 2:** Exposure conditions of the four experiments for estimation of the partition coefficients and diffusion lengths of radon in polymer foils: initial activity concentration of radon $C_{A}$ [MBq/m^3^], exposure duration (sorption time) $t_{s}$ [h], temperature *T* [°C], and the average thickness *L* [µm] of the stack of polymer foils of the given type. The uncertainties are at the level of 1σ. The uncertainties of the thickness include the instrumental uncertainty of the micrometer and the standard deviation of the thickness of the stack of the polymers. “N/A” means that polymers of that type are not used in the given experiment.

$C_{A}$ [MBq/m^3^]	$t_{s}$ [h]	*T* [∘C]	*L* [µm]					
			PP	LDPE	LDPE-A	HDPE	Makrofol N	Makrofol DE
52.4(36)	46.23	21(1)	31.4(11)	74.0(28)	97.0(37)	123.8(18)	42.1(11)	50.6(12)
49.5 (31)	52.03	5(1)	31.1(10)	74.1(24)	92.0(24)	123.8(30)	41.9(11)	50.0(10)
31.4 (20)	48.17	31(1)	29.7(11)	76.7(39)	89.6(11)	120.3(12)	42.0(11)	50.2(11)
1.442(75)	69.43	10(1)	N/A	N/A	N/A	N/A	41.6(11)	50.7(11)

PP – Polypropylene, LDPE – Low-Density Polyethylene, LDPE-A – Low-Density Polyethylene with Anti-slip coating, HDPE – High-Density Polyethylene.

**Table 3:** Partition coefficients polymer–air, diffusion lengths, diffusion coefficients and permeabilities of radon for the studied polymers at different temperatures. The temperature was kept constant within 1 °C. All uncertainties are at the level of 1σ. For comparison, values obtained in previous studies are given.

	PP	LDPE	LDPE-A	HDPE	Makrofol N	Makrofol DE	CD/Makrofol ^a^
*T* [°C]	Partition Coefficient *K*						
5	6.13(55)	4.18(39)	4.05(42)	3.63(33)	211(16)	77.5(67)	21.5(43)
10	–	–	–	–	183(12)	72.8(58)	24.3(36)
21	3.69(38)	3.66(38)	3.13(41)	2.51(22)	103.3(79)	34.6(30)	26.2(19)
31	3.25(43)	3.70(43)	2.96(30)	2.44(21)	70.2(51)	27.8(24)	22.9(10)
20		2.17(14) ^b^ 2.40(22) ^b^		2.21(13) ^b^	112(12) ^c^	27.6(16) ^b^	
*T* [°C]	Diffusion Length $L_{D}$ [µm]						
5	67.6(51)	605(30)	646(36)	460(19)	18.0(10)	20.8(10)	42.2(16)
10	–	–	–	–	23.9(10)	26.8(10)	42.8(11)
21	198(10)	1210(64)	1204(85)	880(22)	36.2(10)	43.3(13)	53.8(5)
31	300(15)	1880(140)	1722(54)	1252(23)	52.1(15)	62.9(16)	75.5(8)
20		1463(33) ^b^ 1437(94) ^b^		721(9) ^b^	38.9(13) ^c^	50.8(10) ^b^	
*T* [°C]	Diffusion Coefficient *D* [10^−14^ m^2^/s]						
5	0.96(14)	76.9(77)	87.4(97)	44.3(37)	0.0677(79)	0.0911(84)	
10	–	–	–	–	0.120(10)	0.151(11)	
21	8.20(85)	307(33)	304(43)	162(8)	0.275(15)	0.394(25)	
31	18.9(19)	739(111)	623(39)	329(12)	0.570(32)	0.831(43)	
*T* [°C]	Permeability *P* [10^−13^ m^2^/s]						
5	0.59(10)	32.1(44)	35.4(54)	16.1(20)	1.43(20)	0.706(89)	
10	–	–	–	–	2.20(24)	1.10(12)	
21	3.03(44)	113(17)	95.1(18)	40.7(41)	2.84(27)	1.36(15)	
31	6.1(10)	273(52)	184(22)	80.4(75)	4.00(37)	2.31(23)	

^a^ Values for Compact Discs (CD) and Makrofol foils reported in [26] (should be compared to Makrofol DE). ^b^ Values for LDPE, HDPE and Makrofol foil (same as ^(a)^) reported in [30]. ^c^ Values for Makrofol N reported in [23]. PP – Polypropylene, LDPE – Low-Density Polyethylene, LDPE-A – Low-Density Polyethylene with Anti-slip coating, HDPE – High-Density Polyethylene.

**Table 4:** Parameters of the linear fits applied to the experimental data shown in Figure 5, Figure 6 and Figure 7, respectively.

	$\ln\left(D\right)=a_{D}+b_{D}T$ Figure 5		$\ln\left(K\right)=a_{K}+b_{K}T$ Figure 6		$\ln\left(P\right)=a_{P}+b_{P}T$ Figure 7	
Polymer	$a_{D}$	$b_{D}$	$a_{K}$	$b_{K}$	$a_{P}$	$b_{P}$
PP	−32.76(35)	0.1159(51)	1.93(11)	−0.0262(59)	−30.87(23)	0.092(10)
LDPE	−28.33(16)	0.0869(80)	1.45(11)	−0.0053(56)	−26.88(19)	0.0815(96)
LDPE−A	−28.13(16)	0.0755(64)	1.45(12)	−0.0123(56)	−26.69(19)	0.0635(82)
HDPE	−28.81(13)	0.0771(55)	1.33(12)	−0.0158(56)	−27.49(16)	0.0619(68)
Makrofol N	−35.22(12)	0.0791(57)	5.603(82)	−0.0441(43)	−29.61(13)	0.0347(59)
Makrofol DE	−35.00(11)	0.0844(54)	4.62(14)	−0.0443(73)	−30.39(14)	0.0410(69)

PP – Polypropylene, LDPE – Low-Density Polyethylene, LDPE-A – Low-Density Polyethylene with Anti-slip coating, HDPE – High-Density Polyethylene.

## Abbreviations

The following abbreviations are used in this manuscript:

LDPE	Low-Density Polyethylene
LDPE-A	Low-Density Polyethylene with Anti-slip coating
HDPE	High-Density Polyethylene
PE	Polyethylene
PP	Polypropylene
LS	Liquid Scintillation
HPGe	High-Purity Germanium
TDCR	Triple to Double Coincidence Ratio
RNG	Radioactive Noble Gas
SLP	Short-Lived Progeny
CD	Compact Disc
SI	International System of Units (from French: Système International (d’unités));
“radon”	short for the ^222^Rn isotope
“thoron”	short for the ^220^Rn isotope
